# Supplementary material for: Multicolor Luminescent Carbon Dots: Tunable Photoluminescence, Excellent Stability, and Their Application in Light-Emitting Diodes
Source: Nanomaterials (Basel). 2022 Sep 9;12(18):3132. doi: 10.3390/nano12183132 (PMC9503501; doi:10.3390/nano12183132)
Supplement: Supplementary file 1 [file nanomaterials-12-03132-s001.zip › nanomaterials-1892455-supplementary.pdf]

Supplementary Materials

Multicolor Luminescent Carbon Dots: Tunable Photoluminescence, Excellent Stability, and their Application in Light-Emitting Diodes

Longshi Rao [1,2,*], Qing Zhang [1,2], Bin Sun [1,2], Mingfu Wen [1,2], Jiayang Zhang [1,2], Guisheng Zhong [1,2], Ting Fu [3] and Xiaodong Niu [1,2]

1 Department of Mechanical Engineering, College of Engineering, Shantou University, Shantou 515063, China

2 Intelligent Manufacturing Key Laboratory of Ministry of Education, Shantou University,
Shantou 515063, China

3 Hubei Key Laboratory of Mechanical Transmission and Manufacturing Engineering, Wuhan University of Science and Technology, Wuhan 430081, China

* Correspondence: lsrao@stu.edu.cn; Tel.: +86-19865045018

Table S1. The reagents and solvents were used to prepare each sample.

Samples	Ammonium citrate (g)	H_2O (mL)	DMF (mL)	Time (Hours)	Temperature (°C)	Typical CDs
0D	3.9	50	0	8	200	B-CDs
5D	3.9	45	5	8	200	
10D	3.9	40	10	8	200	
15D	3.9	35	15	8	200	
20D	3.9	30	20	8	200	G-CDs
25D	3.9	25	25	8	200	
30D	3.9	20	30	8	200	Y-CDs
35D	3.9	15	35	8	200	
40D	3.9	10	40	8	200	
45D	3.9	5	45	8	200	R-CDs
50D	3.9	0	50	8	200	
